# Can we better understand severe mental illness through the lens of Syndemics?

**DOI:** 10.3389/fpsyt.2022.1092964

**Published:** 2023-01-06

**Authors:** Silke Vereeken, Emily Peckham, Simon Gilbody

**Affiliations:** ^1^Mental Health and Addiction, Health Sciences, University of York, York, United Kingdom; ^2^Hull York Medical School, York, United Kingdom

**Keywords:** Syndemics, SMI, MDD, schizophrenia, bipolar disorder, physical activity, smoking, mental health

## Abstract

Current health care systems do not sufficiently address contributors, also known as modifiable behavior factors, to severe mental illnesses (SMI). Instead treatment is focused on decreasing symptom-experience rather than reducing the detrimental effect of biological predisposition and behavioral influences on illness. Health care services and patients alike call for a more comprehensive, individual approach to mental health care, especially for people with SMI. A *Syndemics* framework has been previously used to identify ecological and social contributors to an HIV epidemic in the 1990s, and the same framework is transferable to mental health research to identify the relationship between contributing factors and the outcomes of SMI. Using this approach, a holistic insight into mental illness experience could inform more effective health care strategies that lessen the burden of disease on people with SMI. In this review, the components of a Syndemic framework, the scientific contributions to the topic so far, and the possible future of mental health research under the implementation of a Syndemic framework approach are examined.

## 1. Introduction

The term “Syndemic” has begun to be used to describe synergistic interactions between two or more co-occurring epidemics. It is most commonly used to provide a conceptual framework to understand the multiple causes and consequences of communicable and non-communicable diseases in a socioeconomic context, but has not yet been applied in the context of severe mental illnesses like bipolar disorder, psychosis spectrum disorder, and severe depression. Here we provide an overview of the theory of Syndemics and explore how this might be applied to understand and respond to the most severe forms of mental ill-health, where profound health inequalities and a complex interplay of environmental, behavioral and psychological drivers influence an individual’s health.

The frailty of healthcare systems and their ability to respond to increased demand and widening health inequalities is not a novel problem (1–3). Primary care physicians experience an increase in healthcare seekers, and secondary care facilities (hospitals, psychological wellbeing services) and tertiary care providers (crisis resolution teams) are under a lot of stress to provide resources and allocate help to the increase in demand (3). Because of this, the general population and health care professionals reported suffering from mental ill-health, with an increased incidence of burn-out, mood and anxiety related symptoms and overall greater need for mental health care (4, 5). Mental ill-health is severely detrimental to an individuals’ quality of life, and people with severe mental illnesses (SMIs) like schizophrenia, bipolar disorder, or severe forms of major depressive disorder (MDD) often report a decline in their overall wellbeing and feeling less control over modifiable health behaviors.

People with an SMI suffer from psychological problems that greatly interfere with their ability to engage in functional and occupational activities (6), and frequently report poor physical health and one or more additional, co-occurring illness diagnoses of physical or psychological origin (7). Rates of health risk behaviors, such as smoking, poor diet and low levels of physical activity are very common. Many of these are potentially modifiable. Modifiable health behaviors are often not a choice but the restricted result of the socio-psychological, physical, or environmental impact of living with an SMI or the treatment for it (8). The National Institute of Mental Health defines SMI as non-organic psychosis, marked by a prolonged duration of hospitalization or treatment and atypical social behaviors, impaired work and non-work situations, and an inability to act upon their basic needs (9). No standard definition of SMI in research or in clinical practice exists; many different and equally valid definitions of what constitutes SMI are used, and the general overlap describes severe mental illnesses as any psychiatric diagnosis with symptoms occurring and interfering with daily (healthy) life for at least two years (10). In this paper, we define SMI as schizophrenic spectrum disorder, bipolar and related disorders (Bipolar 1, Bipolar 2, and cyclothymia), and severe MDD.

People with SMI suffer from an increase of symptoms and illness severity when left untreated, especially over longer periods of time (11). To prevent long waiting times for individuals with SMI during which symptoms aggravate to a level that people qualify for emergency care, it is necessary to work on providing more efficient mental health care at both primary and secondary care levels. However, to provide new and efficient care measures it’s essential to know which factors in individuals’ lives contribute to SMI or exacerbate symptoms. Previous research into contributors to SMI has focused on singular contributors to the illnesses (12, 13), and neglected the reality that individuals live complicated lives and have to deal with multiple possible contributing factors to their (mental) health, which can exacerbate or alleviate each other. In the modern world, there is rarely only one aspect of a person’s life that contributes to their mental or overall health, more often it’s an accumulation of different factors from different parts of their lives that either add to or lessen an individual’s (mental) health state. Medical anthropologist Merrill Singer first described this phenomenon of co-occurring and interrelated illness symptoms in epidemics as a *Syndemic*.

## 2. What are Syndemics

The term Syndemic and the underlying theory was first proposed by medical anthropologist Merrill Singer in 1992 (14, 15) to describe the synergistic (interacting, resulting in an enhanced combined effect) interactions between two or more co-occurring diseases or epidemics. The stress of experiencing these co-occurring detrimental contributors can then lead to excess burden of disease experience (16). Synergistic interactions were first introduced to describe the interaction of simultaneously occurring diseases and disease factors and the promoting factors within an individual’s social environment which enhanced the negative effects of disease interaction around the HIV epidemic in drug users in the late 1990s (14). Singer also introduced the notion of “Syndemic vulnerability” to describes the extent to which individual experiences affect co-occurring social and health problems, morbidity and mortality because of the specific eco-psychosocial context of the individual. Syndemic interactions are co-occurring social and health conditions which exacerbate health condition, and includes social-psychological, social-biological, and psychological-biological interactions. By applying the Syndemic approach, pathways can be identified through which certain diseases tend to cluster together. It takes geographical, social, and environmental factors of the co-occurring health conditions into account (17–20). The Framework of Syndemics aims to distribute highly effective strategies that demonstrate how social, political, ecological, and behavioral factors contribute to and increase structural vulnerabilities which lead to adverse disease interaction: a Syndemic (21).

## 3. Syndemic contributors to mental and physical illness in literature

While Syndemics research originated in medical anthropology (22), it has since made its way into a variety of sub-disciplines in research, such as environmental research (23) and health policy research (24). A socio-ecological Syndemic contributor has been identified in socio-economic status (SES) in childhood asthma in North American schools (12, 13). Further evidence supports a relationship between developing childhood asthma and childhood exposure to neighborhood violence, another environmental and traumatic factor enabled by environmental, political, and economic factors (25). A significant connection between Black, Asian and Minority Ethnic (BAME) group-status and personal health and/or vulnerability has been found to contribute to negative health outcomes, as well as other Syndemic vulnerability factors, poverty and discrimination (26), which can lead to poor treatment adherence and increasing drug-resistance in diseases, a dangerous phenomenon referred to as antibiotic resistance (27). Current research lacks insight of bio-eco-geosocial illness interaction, which is vital to develop more effective treatment and prevention strategies in health care. As many physical illnesses co-occur with mental illnesses either as a direct consequence or sequentially, it becomes increasingly important to determine Syndemics within mental and physical health care.

Past research on Syndemics has mainly focused on social contributors to physical health in people with HIV, but researchers recently started exploring the potential of Syndemic contributors to mental health. Stress, poverty, discrimination, and other forms of social adversity are the primary route through which social factors have been found to contribute to negative mental health outcomes (28–30). Evidence linked mechanisms at the biological and cellular level are linked to levels of stress, impaired immune system functions, and illness development (31), which has been demonstrated to result in psychoneuroimmunology in individuals (32). Experiencing social stigma can lead to psychological stress in the individual, and evidence for a link between both positive and negative social relationships and mental health status has been published (16). Substantial evidence shows that caregivers of children with developmental disabilities are more susceptible to developing anxiety, depression, and a range of physical symptoms and illnesses (33). High levels of stress were found to be a likely mediator in this Syndemic model (34). A Syndemic model of a cumulative effect of psychiatric morbidity, substance misuse, violence, and poor physical health contributing to reduced life expectancy showed that multiple socioeconomic, political, genetic, and environmental factors increase susceptibility to several life threatening physical illnesses (35). Mental disorders have been shown to lead to a higher prevalence of high-risk behaviors, reduced self-care behaviors, and a decrease in help-seeking (36, 37). An amalgamation of detrimental behaviors increases the risk of developing both communicable and non-communicable physical illnesses, which demonstrates a Syndemic model of violence, psychiatric morbidity, substance abuse, and behavioral/biological physical health risks.

Two pathways explain the theory behind how these Syndemic factors could contribute to an increased chance of premature death (35); one way could be via chronic or acute increased exposure to suicidal ideation, substance abuse, and poor access to healthcare, which generates mental and physical stress. Another way could be via early childhood or young adulthood precursors of more severe physical conditions that result in mortality, like learned detrimental health behaviors, a lack of exercise or poor diet, violence-related trauma, or learned helplessness. Furthermore, a Syndemic model of the influence of felt social isolation and neighborhood characteristics on people with psychosis has been demonstrated (38).

A recent acceleration of interest in factors that could influence mental health has shed more light on the effect of exposure to nature and air pollution exposure on the development of psychopathology. Exposure to ambient air pollution (toxic and harmful emissions by industry, household, and road traffic) has been found to predict individual risk of developing adolescent MDD in the UK and Brazil via systemic inflammation and stress in the body (23). There is furthermore evidence for a significant link between exposure to air pollution early in life and the development of psychopathology (39–41). Outdoor air pollution has been associated with negative outcomes such as adverse changes in the cardiovascular and respiratory systems and harm to the central nervous system (CNS), which has been linked to diverse CNS damage like chronic neuroinflammation, glial-cell dysregulation, vascular damage in global brain structure integrity, and neuron proliferation in children (42–44). This is hypothesized to lead to a decrease in emotional control, difficulties with inhibition, and regulating behavioral and cognitive emotional responses in situations. Living in urban settings has also been associated with adolescent psychotic experiences, and air pollution has explained 60% of that association, with noise pollution being another significant contributor (45). Air pollutants seem to have potent oxidative effects on proteins and lipids affecting the development of the nasal epithelium and blood-brain barrier, and can lead to neuroinflammation and neurodegeneration in the brain regions of the frontal cortex and olfactory bulb (45). This in turn has been found to increase the risk of experiencing psychotic episodes (46) and developing progressive neuronal death, often found in Alzheimer’s and other neurodegenerative diseases (47). Noise pollution has been linked to subclinical psychotic phenomena, moderated by sleep disturbances, stress, and cognitive developmental issues in children and adolescents (45). MDD, psychotic phenomena, and complications from neuroinflammation can all contribute to the development of SMIs, which were found to often be preceded by cognitive development, anxiety, sleep disturbances, affective liability, and/or basic as well as psychotic-like experiences (48). Severe mental illness is known to vary per individual as per their genetic susceptibility, which is supported by the finding that one in three children of parents with SMI are found to develop an MDD or other SMI by young adulthood (49).

While these papers together indirectly demonstrate the possible existence of a Syndemic interaction between socio-geological factors of mental health and physical health, the root causes and pathway developments that exacerbate illness development in individuals is still unclear. There is ample scientific evidence in literature demonstrating that geo-environmental determinants (noise and air pollution) can influence neuronal development and increase the likelihood of developing both physical illness and SMIs. The evidence for multiple specific contributors can arguably be considered evidence for the existence of a Syndemic model of mental and physical health. However, these factors have not yet been investigated in how they Syndemically contribute to the development of SMI and each other, proving a gap in scientific literature. It is vital for future health care efficiency to investigate these specific contributors and their Syndemic effect on SMI development. Furthermore, it is important to investigate the weight and direction of Syndemic contributors in mental illness so health care systems can adequately address these factors in prevention and treatment strategies. The picture of a wide, network-model of mental, physical, and neurocognitive health, as well as environmental determinants all Syndemically interacting and co-occurring presents itself, and future research around determinants of SMI needs to use a Syndemic framework approach to effectively improve the health care people with SMI need to receive.

## 4. Analyzing Syndemics theory in research

Literature around Syndemics has not only struggled to clearly identify a Syndemic in research and research questions, but has recently questioned the common statistical methods used to analyze contributing variables. The methodological issue within Syndemic research that to analyze the impact of Syndemic interactions, researchers have mainly used either regression analyses or ethnographic approaches to show relationships between Syndemic co-factors. Ethnographic analysis methods, usually of a qualitative nature, investigate the interaction between Syndemic contributors (24). While these analysis methods provide insight to social context-specific trends and the possible effects of policies and programs within a Syndemic framework approach to models of ill-health, a call for more nuanced analysis methods to investigate the co-existence of and interaction between drivers of health and disease has been made (24, 50).Using regression analysis methods for Syndemic models has been criticized for reducing the weight of the results, because regression analyses with an additive Syndemic (co-)factor does not consider the individual contributing weight of influence in a model as complex as one depicting an eco-psychosocial Syndemic (51–54). Sum scores are mainly a demonstration of the cumulative effect of multiple psychosocial and structural adversities, and neglect the interactive Syndemic element of co-morbidities which exacerbate health outcomes. Some papers have implemented higher level modeling techniques like structural equation modeling or frequencies and descriptives evaluations to provide statistical evidence of synergistic relations between the contributing factors on a health outcome variable (24). Another analysis method previously used in Syndemic framework investigations is the synergy factor (SF) analysis; in SF analysis, binary interactions are investigated for significance and size in case-control studies and meta-analyses, and odds ratios as well as the synergy factor is calculated using logistic regression models based upon these interactions (50).Using social network analysis demonstrated that the number of included Syndemic risk factors both increased the additive component of physical health outcomes, as well as synergistically increasing it (55). The use of disease maps can increase understanding Syndemic interactions between contributors and demonstrate how these are influenced by political decisions within community contexts (56). However, using disease maps to analyze Syndemic interactions requires additional contextual analysis and interpreting the origin and size of the dataset. More guidelines need to be given with regards to where to collect data for representative Syndemic research, whether it is sufficient in size, and how potential Syndemic relationships can be extracted, if it is to be used as a standard for evaluating Syndemic frameworks (24). To explain underlying biological and statistical pathways in illness development, a network model of interconnected medical and social contributors to health disparities in the population has been suggested (57). A call for Syndemic models to facilitate integration of the findings into health care by improving the methods of analyses conducted in current research has been made (54). Expanding analyses methods to include both individual and population levels in a multilevel analytical approach, and thereby researching distal Syndemic contributors, could lead to discovering more impactful and efficient interventions and preventive measures to improve overall health in vulnerable populations (52). To analyze multiple level data, structural equation modeling (50, 58), multivariate multilevel regression models (59), or multilevel logistic modeling (60) analyses can be conducted.

In sum, little evidence has been found about how contributing factors in Syndemic models actually exercise their effects, because the way to analyze data has been inconsistent. Other than evidence for there being *an* interaction between independent factors which all contribute to the outcome, no currently published research has investigated *how* these interactions take place. Furthermore, research regarding biopsychological interactions has so far focused on investigating the mediating or moderating roles of singular factors, not synergistic relationships. Earlier research fails to position the many contributing factors to SMI and physical illnesses within the bigger sociobiological and socio-economic picture in communities, and therefore only captured part of the disease picture. This can be attributed to there not being standardized analysis methods or guideline for Syndemic framework research. And while researchers generally call for further exploration of the relationships within these models to aid the development of a better and more efficient health care system, very little research has actually been published to do just that. Interacting, Syndemic contributors to physical illness and SMI are more commonly investigated on their own and not in how their interactions with each other influence the development of SMIs. The need for an integrative approach to analyzing all Syndemic contributors within a Syndemic framework is evident.

## 5. How might mental health care and policy embrace a Syndemic Framework?

It becomes clear therefore that Syndemics research, when correctly applied to investigating the relationship between factors and outcomes, could make a significant contribution to modern health care structures. From the beginning of Syndemic research, the need for better assessments of Syndemic contributors within the public health system and social communities has been stressed (16). Both sectors need to better monitor proven factors that contribute to physical ill-health or illness clusters, and enforce early medical and public health responses in the face of Syndemically interacting health epidemics more strongly. Doing so would lead to a more integrated approach to health care on a community-level by improving contributors and lessening their detrimental impact on mental health at an early stage of illness development. A Syndemic framework approach could provide health care systems with the necessary help to implement findings toward patient-care at a preventive stage rather than an interventional stage, which could decrease the need for urgent mental health care.

People with an SMI experience a significant health and mortality gap (61), the implementation of a Syndemic framework could aid in developing health care strategies to reduce this gap. Three ways in which implementing a Syndemics framework could advance public health, medicine and human rights have been stated (62): First, Syndemic knowledge provides information which makes interventions on policy and clinical levels more efficient. Second, a Syndemic framework could improve general understanding of the specific factors that make some communities more vulnerable to lower social and biological wellbeing. And third, implementing a Syndemic construct could fuel strategies for recognizing which socio-political-ecological factors create and perpetuate structural vulnerabilities that facilitate Syndemic exacerbation and emergence. As of now, health care systems can only address contributors to SMI one by one even if multiple contributors are known to interact and even exacerbate each other.

By placing more focus on early prevention and intervention strategies in general communities, specific primary and secondary (mental) health care interventions could be developed to decrease the severity of symptoms in (severe) mental illness, and less people would suffer from the long waitlists and delayed treatment availability. Health care has become increasingly hard to access due to extensive waiting lists, exacerbated by overworked personnel and staff (2). The high workload and pressure on (mental) health care staff has intensified over the past 20 years, seeing a steep increase in emergency and urgent care requests for psychiatrists and mental health services (5). Unsurprisingly, health care staffs also suffer from burn-out-related symptoms, mood and anxiety illnesses, and even post-traumatic stress disorders (PTSD) in a significant number (3). The bandwidth of (mental) health care services and staff seems even more deflated and depleted as a natural consequence of the increase in workload. The health care sector has reported shortages globally (1). This constant shortage of staff increases stress and pressure on individual and communal health carers in charge of the wellbeing of help-seekers. Expanding the amount of health care roles in different levels of prevention and intervention could decrease the stress and responsibility put on one single role within the sector, and create for a broader range of health care provision, an increase in appeal to work in the sector, and a better overall understanding of what contributes to health for the general population. For example, the integration of community health (social) workers to improve patients’ adherence to treatment even when faced with structural vulnerabilities and a lack of self-motivation could eventually lead to a stronger, more accessible, and more affordable health care system for vulnerable communities (62, 63). Public health community nurses could also take interconnected social and medical factors that influence health disparities within the specific neighborhoods into consideration, and contributors to epidemics (and finally, Syndemics) would be more likely to be caught and treated early on (57). Health nurses could counteract contributing factors of a Syndemic in primary prevention (applying information about Syndemic components), secondary prevention (acknowledging and addressing detrimental factors), and tertiary prevention strategies (community correction programs, community mental health programs (57). To do so, Syndemic components to (severe mental) illnesses and illness development and maintenance need to be identified in a collective context.

In conclusion, implementing a Syndemic framework could have a great impact on clinical health care both for prevention and intervention measures, because Syndemically informed interventions address the roots of inequalities in patient care. Syndemically informed interventions could strengthen and improve prevention strategies by incorporating the full scope of complex contexts in which Syndemic vulnerabilities occur holistically. Furthermore, the implementation of a Syndemic framework in (mental) health care would lead to the creation of more health care roles at differing levels of prevention/intervention, which would decrease the pressure and workload put on health care workers. More Syndemic research into contributors to SMI is therefore needed to improve the current health care system and approach.

## 6. The future of Syndemic research in mental health

**FIGURE 1:**
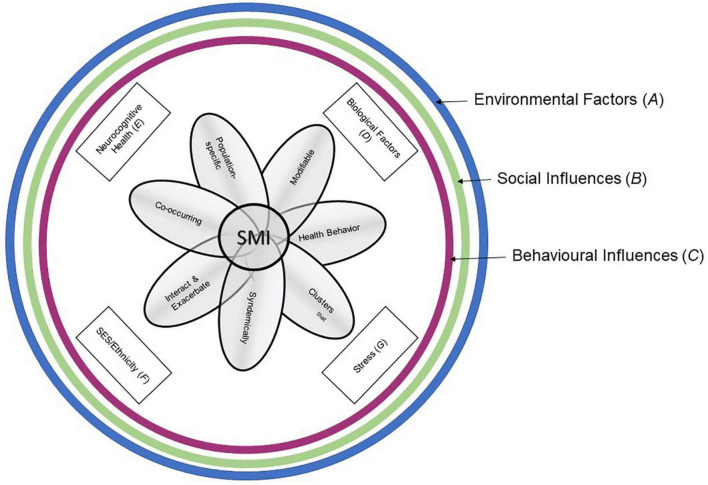
Syndemic Framework to SMI Development and Maintenance. The figure shows the inward trajectory of how influential layers of habits and modifiable behaviors can contribute to the course of an SMI in individuals. Broader environmental factors influence social factors, which in turn influence common behaviors. The more individual contributors of biological factors, neurocognitive health, SES/Ethnicity status, and stressors, influenced also by the greater factors encircling them, lead to a more nuanced and person-specific picture of SMI development and maintenance, and in turn influence specific modifiable behaviors with a direct impact on an individual’s SMI.

As the research discussed in this manuscript has suggested, implementing Syndemic theory and frameworks into current health care treatment could significantly impact mental and physical wellbeing in disadvantaged communities and individuals who experience the effect of detrimental contributors to their overall wellbeing. Based on previously published literature on individual contributors to mental illness, we developed a model of the factors that contribute to SMI development and maintenance (Figure 1). This model demonstrates the multilevel influence of contributors to SMI in individuals, and the nuancing trajectory from broad, common outside influences to specific modifiable health behaviors that affect the development and maintenance of (severe) mental illness. Individuals aren’t born with fully developed SMI, there is a gradual increase in symptoms and decline in wellbeing (64). Early screening and intervention, which often also include a lower threshold to both request and follow health care help plans, would decrease the amount of people requiring urgent help long-term. But specific Syndemic contributors to SMIs need to be investigated to give an applicable, holistic picture of what these screenings and interventions could look like.

In addition to the environmental and sociobiological factors discussed earlier on, modifiable health behaviors and behavior changes could have a synergistic effect on the development of SMIs, as they have already been proven to have an effect on the development of SMIs individually or in mediation/moderation models. In mental health care, treatment adherence is often facilitated or impeded by self-motivation (63), self-efficacy and personal resilience. Personal resilience, which refers to an individual’s ability to bounce back from exposure to severe risk or distress, has been shown to lead to a more stable state of mind, better mental health, and increased tenacity to overcome obstacles, but generally becomes depleted with repeated exposure to negative circumstances and influencing factors (65–67). Many factors have been established to contribute to personal resilience, for example experienced loneliness (68), levels of physical activity (69), felt social support (70), and perceived in-group belonging (71). Early community interventions based on Syndemic knowledge and aimed toward improving individual and group resilience could provide people with the necessary skills and knowledge to improve and maintain good resilience and therefore better mental health and overall wellbeing. Future research into Syndemic contributors to SMIs needs to take personal resilience into account and investigate its potential role in a Syndemically framed health care system.

The effect of low physical activity on physical and mental health has been demonstrated, and low physical activity levels have been associated with the development and maintenance of several SMIs (72, 73). However, levels of physical activity have not yet been included in a Syndemic model approach to SMIs in the population, and therefore not all its Syndemic effects in the bigger picture of illness development have been established. The exact pathways in with physical activity Syndemically affects SMI development need to be investigated in future research.

Likewise, smoking behavior can partly explain the mortality rate in people with schizophrenia, and overall worsen symptom experience for people with SMI (74). Research shows that an increase in smoking behavior is related to an increase in negative symptom experience (75), but smoking behavior also influences other modifiable behaviors like physical activity levels, and sociobiological processes (76). The effect of smoking behavior in a Syndemic model of SMI development and modifiable health behaviors has not yet been investigated, but as there is a reciprocal influence between this health behavior and others previously mentioned, it is important to include smoking behavior in future research on the Syndemic effects in SMI development.

Another interesting contributor to the development of SMIs is exposure to nature. Recent papers have established a relationship between the amount of time spent in natural surroundings and green environments, and the experience of mental health outcomes (77). Exposure to natural and green spaces is distinctly different from air and noise pollution, which has been demonstrated to have a significant Syndemic effect on developing SMI as previously discussed. While a relationship between the contributor and outcome has been established, the pathways in which this effect takes place have not yet been investigated, nor interrelationships to other known contributors to (severe) mental illness. Furthermore, because certain symptoms of SMI and living in urban settings (78) can make it difficult to venture into green or natural spaces, the possibility of a Syndemic model of the relationship between exposure to nature and SMI experience and development begs investigation.

Syndemic knowledge and how to implement it is essential for a better future in treating severe mental illness, overcoming community-wide detrimental circumstances, and improving overall wellbeing in people with, but also without SMIs. More research needs to be done to identify Syndemic contributors in the form of modifiable health behaviors that affect SMI development and severity in the population as well as their underlying pathways, and there needs to be more evidence on which modifiable health behaviors treated with community-level interventions could improve the quality of health care interventions implemented in the future. Early, Syndemically framed interventions based on the model of Syndemic contributors to SMI suggested in this paper could improve overall mental wellbeing, quality of life, and increased recovery for people with mental illness. The aim of future research into Syndemics in mental health should be to inform the development of primary, community-based, Syndemically informed interventions aimed at modifiable health behaviors to tackle health care inequalities among health care seekers with mental illness.

## Data availability statement

The original contributions presented in this study are included in the article/supplementary material, further inquiries can be directed to the corresponding author.

## Author contributions

SV created the general body of the review. EP and SG supplied and enhanced the body of text and the emerged figure with edits and suggestions, and approved the final version. All authors contributed to the article and approved the submitted version.

## References

[1] Tsolekile L Abrahams-Gessel S Puoane T. Healthcare professional shortage and task-shifting to prevent cardiovascular disease: implications for low- and middle-income countries. *Curr Cardiol Rep.* (2015) 17:115. 10.1007/s11886-015-0672-y 26482758 PMC13718040

[2] Sant’Ana G Imoto A Amorim F Taminato M Peccin M Santana L Infection and death in healthcare workers due to COVID-19: a systematic review. *Acta Paul Enferm.* (2020) 33:2–6. 10.37689/acta-ape/2020AO0107

[3] Chirico F Ferrari G Nucera G Szarpak L. Prevalence of anxiety, depression, burnout syndrome, and mental health disorders among healthcare workers during the COVID-19 pandemic: a rapid umbrella. *J Health Soc.* (2021). Available from: https://journalhss.com/wp-content/uploads/jhhs_62_209-220.pdf (accessed February 21, 2022).

[4] Veldhuis C Nesoff ED McKowen A Rice D Ghoneima H Wootton A Addressing the critical need for long-term mental health data during the COVID-19 pandemic: changes in mental health from April to September 2020. *Prev Med.* (2021) 146:106465. 10.1016/j.ypmed.2021.106465 33647353 PMC8136863

[5] Rcpsych. *Psychiatrists See Alarming Rise in Patients Needing Urgent and Emergency Care and Forecast a “Tsunami” of Mental Illness.* (2020). Available online at: https://www.rcpsych.ac.uk/news-and-features/latest-news/detail/2020/05/15/psychiatrists-see-alarming-rise-in-patients-needing-urgent-and-emergency-care (accessed February 24, 2022).

[6] De Hert M Correll C Bobes J Cetkovich-Bakmas M Cohen D Asai I Physical illness in patients with severe mental disorders. I. Prevalence, impact of medications and disparities in health care. *World Psychiatry.* (2011) 10:52–77. 10.1002/j.2051-5545.2011.tb00014.x 21379357 PMC3048500

[7] Heller T Roccoforte J Hsieh K Cook J Pickett S. Benefits of support groups for families of adults with severe mental illness. *Am J Orthopsychiatry.* (1997) 67:187–98. 10.1037/h0080222 9142352

[8] Robson D Gray R. Serious mental illness and physical health problems: a discussion paper. *Int J Nurs Stud.* (2007) 44:457–66. 10.1016/j.ijnurstu.2006.07.013 17007859

[9] NIMH. *Towards a Model for a Comprehensive Community-Based Mental Health System.* Washington, DC: NIMH (1987).

[10] Wiersma D. Needs of people with severe mental illness. *Acta Psychiatr Scand Suppl.* (2006) 113:115–9. 10.1111/j.1600-0447.2005.00728.x 16445493

[11] Medeiros G Senço S Lafer B Almeida K. Association between duration of untreated bipolar disorder and clinical outcome: data from a Brazilian sample. *Braz J Psychiatry.* (2016) 38:6–10. 10.1590/1516-4446-2015-1680 26785105 PMC7115469

[12] Schwab N Cullen M Schwartz J. A survey of the prevalence of asthma among school age children in Connecticut. *Environ Hum Health Incorp.* (2000).

[13] Miller J. The effects of race/ethnicity and income on early childhood asthma prevalence and health care use. *Am J Public Health.* (2000) 90:428–30. 10.2105/AJPH.90.3.428 10705865 PMC1446167

[14] Singer M. A dose of drugs, a touch of violence, a case of AIDS: conceptualizing the sava syndemic. *Free Inq Creat Sociol.* (2000) 28:13–24.

[15] Singer M Snipes C. Generations of suffering: experiences of a treatment program for substance abuse during pregnancy. *J Health Care Poor Underserved* (1992) 3:222–34; discussion 235–9. 10.1353/hpu.2010.0180 1327239

[16] Singer M Clair S. Syndemics and public health: reconceptualizing disease in bio-social context. *Med Anthropol Q.* (2003) 17:423–41. 10.1525/maq.2003.17.4.423 14716917

[17] Alcabes P Schoenbaum E Klein R. Correlates of the rate of decline of CD4+ lymphocytes among injection drug users infected with the human immunodeficiency virus. *Am J Epidemiol.* (1993) 137:989–1000. 10.1093/oxfordjournals.aje.a116771 8100395

[18] Ensoli F Sirianni M. HIV/HCV co-infection: clinical and therapeutic challenges. *AIDS.* (2002) 16:1419–20. 10.1097/00002030-200207050-00014 12131219

[19] Farizo K. Spectrum of disease in persons with human immunodeficiency virus infection in the United States. *JAMA.* (1992) 267:1798. 10.1001/jama.1992.03480130114035 1347573

[20] Rose A Sinka K Watson J Mortimer J Charlett A. An estimate of the contribution of HIV infection to the recent rise in tuberculosis in England and Wales. *Thorax.* (2002) 57:442–5. 10.1136/thorax.57.5.442 11978923 PMC1746328

[21] Peprah E Caler E Snyder A Ketema F. Deconstructing syndemics: the many layers of clustering multi-comorbidities in people living with HIV. *Int J Environ Res Public Health.* (2020) 17:4704. 10.3390/ijerph17134704 32629920 PMC7369980

[22] Duby Z Jonas K Appollis T Maruping K Dietrich J Vanleeuw L “There is no fear in me …well, that little fear is there”: dualistic views towards HIV testing among South African adolescent girls and young women. *Afr J AIDS Res.* (2020) 19:214–21. 10.2989/16085906.2020.1799232 32892703

[23] Latham R Kieling C Arseneault L Botter-Maio Rocha T Beddows A Beevers S Childhood exposure to ambient air pollution and predicting individual risk of depression onset in UK adolescents. *J Psychiatr Res.* (2021) 138:60–7. 10.1016/j.jpsychires.2021.03.042 33831678 PMC8412033

[24] Mendenhall E Singer M. What constitutes a syndemic? Methods, contexts, and framing from 2019. *Curr Opin HIV AIDS.* (2020) 15:213–7. 10.1097/COH.0000000000000628 32412998

[25] Wright R Hanrahan J Tager I Speizer F. Effect of the exposure to violence on the occurrence and severity of childhood asthma in an inner-city population. *Am J Respir Crit Care Med.* (1997) 155:A972.

[26] Willen S Knipper M Abadía-Barrero C Davidovitch N. Syndemic vulnerability and the right to health. *Lancet.* (2017) 389:964–77. 10.1016/S0140-6736(17)30261-1 28271847

[27] CDC. *Antibiotic Resistance Threats in the United States, 2019.* Washington, DC: US Department of Health and Human Services (2019).

[28] Blakey M. *Psychophysiological Stress and. Diagnosing America: Anthropology and Public Engagement.* (1994). p. 149. Available online at: https://books.google.com/books?hl=en&lr=&id=5WAKK1P7EBYC&oi=fnd&pg=PA149&dq=Blakey,+M.L.,+1994.+Psychophysiological+stress+and+disorders+of+industrial+society:+a+critical+theoretical+formulation+for+biocultural+research.+Diagnosing+America:+Anthropology+and+public+engagement&ots=t686ANjzYS&sig=6avbkz0EBptinO8elFYTZbb6xbE (accessed January 27, 2022).

[29] Dressler W. Culture, stress, and cardiovascular disease. *Encycloped Med Anthropol.* (2003) 328–34. 10.1007/0-387-29905-X_39

[30] McDade T. Status incongruity in Samoan youth: a biocultural analysis of culture change, stress, and immune function. *Med Anthropol Q.* (2002) 16:123–50. 10.1525/maq.2002.16.2.123 12087626

[31] Cardinali D. Book review psychoneuroimmunology. *N Engl J Med.* (2001) 344:695–6. 10.1056/NEJM200103013440922

[32] Miller G Cohen S. Psychological interventions and the immune system: a meta-analytic review and critique. *Health Psychol.* (2001) 20:47–63. 10.1037//0278-6133.20.1.47 11199066

[33] Pickett K Ajebon M Hou B Kelly B Bird P Dickerson J Vulnerabilities in child wellbeing among primary school children: a cross-sectional study in Bradford, UK. *medRxiv* [Preprint]. (2021). Available online at: http://medrxiv.org/lookup/doi/10.1101/2021.01.10.21249538 (accessed February 4, 2022). 10.1136/bmjopen-2021-049416 PMC9247690 35772827

[34] Masefield S Prady S Sheldon T Small N Jarvis S Pickett K. The effects of caring for young children with developmental disabilities on mothers’ health and healthcare use: analysis of primary care data in the born in Bradford cohort. *J Dev Phys Disabil.* (2021) 34:67–87. 10.1007/s10882-021-09789-7

[35] Coid J Zhang Y Bebbington P Ullrich S de Stavola B Bhui K A syndemic of psychiatric morbidity, substance misuse, violence, and poor physical health among young Scottish men with reduced life expectancy. *SSM Popul Health.* (2021) 15:100858. 10.1016/j.ssmph.2021.100858 34307825 PMC8258690

[36] Singer M Bulled N Ostrach B Mendenhall E. Syndemics and the biosocial conception of health. *Lancet.* (2017) 389:941–50. 10.1016/S0140-6736(17)30003-X 28271845

[37] Pantalone D Nelson K Batchelder A Chiu C Gunn H Horvath KJ. A systematic review and meta-analysis of combination behavioral interventions co-targeting psychosocial syndemics and HIV-related health behaviors for sexual minority men. *J Sex Res.* (2020) 57:681–708. 10.1080/00224499.2020.1728514 32077326 PMC7457381

[38] Giacco D Kirkbride J Ermakova A Webber M Xanthopoulou P Priebe S. Neighbourhood characteristics and social isolation of people with psychosis: a multi-site cross-sectional study. *Soc Psychiatry Psychiatr Epidemiol.* (2021) 57:1907–15. 10.1007/s00127-021-02190-x 34791516 PMC9375739

[39] Klompmaker J Janssen N Bloemsma L Marra M Lebret E Gehring U Effects of exposure to surrounding green, air pollution and traffic noise with non-accidental and cause-specific mortality in the Dutch national cohort. *Environ Health.* (2021) 20:82. 10.1186/s12940-021-00769-0 34261495 PMC8281461

[40] Rocha T Fisher H Caye A Anselmi L Arseneault L Barros F Identifying adolescents at risk for depression: a prediction score performance in cohorts based in 3 different continents. *J Am Acad Child Adolesc Psychiatry.* (2021) 60:262–73. 10.1016/j.jaac.2019.12.004 31953186 PMC8215370

[41] Reuben A Arseneault L Beddows A Beevers S Moffitt T Ambler A Association of air pollution exposure in childhood and adolescence with psychopathology at the transition to adulthood. *JAMA Netw Open.* (2021) 4:e217508. 10.1001/jamanetworkopen.2021.7508 33909054 PMC8082321

[42] Herting M Younan D Campbell C Chen J. Outdoor air pollution and brain structure and function from across childhood to young adulthood: a methodological review of brain MRI studies. *Front Public Health.* (2019) 7:332. 10.3389/fpubh.2019.00332 31867298 PMC6908886

[43] Salvi A Salim S. Neurobehavioral consequences of traffic-related air pollution. *Front Neurosci.* (2019) 13:1232. 10.3389/fnins.2019.01232 31824243 PMC6881276

[44] Costa L Cole T Coburn J Chang Y Dao K Roqué P. Neurotoxicity of traffic-related air pollution. *Neurotoxicology.* (2017) 59:133–9. 10.1016/j.neuro.2015.11.008 26610921 PMC4875879

[45] Newbury J Arseneault L Beevers S Kitwiroon N Roberts S Pariante C Association of air pollution exposure with psychotic experiences during adolescence. *JAMA Psychiatry.* (2019) 76:614–23. 10.1001/jamapsychiatry.2019.0056 30916743 PMC6499472

[46] Calderón-Garcidueñas L Mora-Tiscareño A Fordham L Valencia-Salazar G Chung C Rodriguez-Alcaraz A Respiratory damage in children exposed to urban pollution. *Pediatr Pulmonol.* (2003) 36:148–61. 10.1002/ppul.10338 12833495

[47] Calderón-Garcidueñas L Solt A Henríquez-Roldán C Torres-Jardón R Nuse B Herritt L Long-term air pollution exposure is associated with neuroinflammation, an altered innate immune response, disruption of the blood-brain barrier, ultrafine particulate deposition, and accumulation of amyloid beta-42 and alpha-synuclein in children and young adults. *Toxicol Pathol.* (2008) 36:289–310. 10.1177/0192623307313011 18349428

[48] Uher R Cumby J MacKenzie L Morash-Conway J Glover J Aylott A A familial risk enriched cohort as a platform for testing early interventions to prevent severe mental illness. *BMC Psychiatry.* (2014) 14:344. 10.1186/s12888-014-0344-2 25439055 PMC4267051

[49] Rasic D Hajek T Alda M Uher R. Risk of mental illness in offspring of parents with schizophrenia, bipolar disorder, and major depressive disorder: a meta-analysis of family high-risk studies. *Schizophr Bull.* (2014) 40:28–38. 10.1093/schbul/sbt114 23960245 PMC3885302

[50] Bulled N. A new approach to measuring the synergy in a syndemic: revisiting the SAVA syndemic among urban MSM in the United States. *Glob Public Health.* (2022) 17:2070–80. 10.1080/17441692.2021.1974513 34506253

[51] Tsai A. Syndemics: a theory in search of data or data in search of a theory? *Soc Sci Med.* (2018) 206:117–22. 10.1016/j.socscimed.2018.03.040 29628175 PMC6613368

[52] Tsai A Mendenhall E Trostle J Kawachi I. Co-occurring epidemics, syndemics, and population health. *Lancet.* (2017) 389:978–82. 10.1016/S0140-6736(17)30403-8 28271848 PMC5972361

[53] Tsai A Burns B. Syndemics of psychosocial problems and HIV risk: a systematic review of empirical tests of the disease interaction concept. *Soc Sci Med.* (2015) 139:26–35. 10.1016/j.socscimed.2015.06.024 26150065 PMC4519429

[54] Tsai A Venkataramani A. Syndemics and health disparities: a methodological note. *AIDS Behav.* (2016) 20:423–30. 10.1007/s10461-015-1260-2 26662266 PMC4755906

[55] Scheer J Pachankis J. Psychosocial syndemic risks surrounding physical health conditions among sexual and gender minority individuals. *LGBT Health.* (2019) 6:377–85. 10.1089/lgbt.2019.0025 31644383 PMC6918840

[56] McLuckie C Pho M Ellis K Navon L Walblay K Jenkins W Identifying areas with disproportionate local health department services relative to opioid overdose, HIV and hepatitis C diagnosis rates: a study of rural Illinois. *Int J Environ Res Public Health.* (2019) 16:989. 10.3390/ijerph16060989 30893862 PMC6466434

[57] Kelly P Cheng A Spencer-Carver E Ramaswamy M. A syndemic model of women incarcerated in community jails. *Public Health Nurs.* (2014) 31:118–25. 10.1111/phn.12056 24588130 PMC4260392

[58] Himmelgreen D Romero-Daza N Heuer J Lucas W Salinas-Miranda A Stoddard T. Using syndemic theory to understand food insecurity and diet-related chronic diseases. *Soc Sci Med.* (2022) 295:113124. 10.1016/j.socscimed.2020.113124 32586635

[59] Hox J Moerbeek M van de Schoot R editors. Multilevel analysis. 3rd ed. In: *Quantitative Methodology Series*. New York, NY: Routledge (2017). 10.4324/9781315650982

[60] Sommet N Morselli D. Keep calm and learn multilevel logistic modeling: a simplified three-step procedure using Stata, R, mplus, and SPSS. *Int Rev Soc Psychol.* (2017) 30:203–18. 10.5334/irsp.90

[61] Spanakis P Peckham E Mathers A Shiers D Gilbody S. The digital divide: amplifying health inequalities for people with severe mental illness in the time of COVID-19. *Br J Psychiatry.* (2021) 219:529–31. 10.1192/bjp.2021.56 35048887 PMC8111186

[62] Mendenhall E. Syndemics: a new path for global health research. *Lancet.* (2017) 389:889–91. 10.1016/S0140-6736(17)30602-5 28271827

[63] Solbjør M Rise M Westerlund H Steinsbekk A. Patient participation in mental healthcare: when is it difficult? A qualitative study of users and providers in a mental health hospital in Norway. *Int J Soc Psychiatry.* (2013) 59:107–13. 10.1177/0020764011423464 22013139

[64] Dewa L Cecil E Eastwood L Darzi A Aylin P. Indicators of deterioration in young adults with serious mental illness: a systematic review protocol. *Syst Rev.* (2018) 7:123. 10.1186/s13643-018-0781-y 30115109 PMC6097392

[65] Galli N Vealey R. “bouncing back” from adversity: athletes’ experiences of resilience. *Sport Psychol.* (2008) 22:316–35. 10.1123/tsp.22.3.316

[66] Lu F Lee W Chang Y Chou C Hsu Y Lin J Interaction of athletes’ resilience and coaches’ social support on the stress-burnout relationship: a conjunctive moderation perspective. *Psychol Sport Exerc.* (2016) 22:202–9. 10.1016/j.psychsport.2015.08.005

[67] Wu G Feder A Cohen H Kim J Calderon S Charney D Understanding resilience. *Front Behav Neurosci.* (2013) 7:10. 10.3389/fnbeh.2013.00010 23422934 PMC3573269

[68] Gerino E Rollè L Sechi C Brustia P. Loneliness, resilience, mental health, and quality of life in old age: a structural equation model. *Front Psychol.* (2017) 8:2003. 10.3389/fpsyg.2017.02003 29184526 PMC5694593

[69] Moljord I Moksnes U Espnes G Hjemdal O Eriksen L. Physical activity, resilience, and depressive symptoms in adolescence. *Ment Health Phys Act.* (2014) 7:79–85. 10.1016/j.mhpa.2014.04.001

[70] Sippel L Pietrzak R Charney D Mayes L Southwick S. How does social support enhance resilience in the trauma-exposed individual? *Ecol Soc.* (2015) 20:10. 10.5751/ES-07832-200410 30174746

[71] Scarf D Moradi S McGaw K Hewitt J Hayhurst J Boyes M Somewhere I belong: long-term increases in adolescents’ resilience are predicted by perceived belonging to the in-group. *Br J Soc Psychol.* (2016) 55:588–99. 10.1111/bjso.12151 27448617

[72] Vancampfort D Kimbowa S Ward P Mugisha J. Physical activity, physical fitness and quality of life in outpatients with a psychotic disorder versus healthy matched controls in a low-income country. *Schizophr Res.* (2021) 229:1–2. 10.1016/j.schres.2021.01.019 33607606

[73] McGuire D Shannon A Somaiya J Brown E O’Donoghue B. A pilot study of a yoga intervention for the treatment of anxiety in young people with early psychosis. *Early Interv Psychiatry.* (2022) 16:200–4. 10.1111/eip.13151 33929083

[74] Brown S Inskip H Barraclough B. Causes of the excess mortality of schizophrenia. *Br J Psychiatry.* (2000) 177:212–7. 10.1192/bjp.177.3.212 11040880

[75] Snyder M McDevitt J Painter S. Smoking cessation and serious mental illness. *Arch Psychiatr Nurs.* (2008) 22:297–304. 10.1016/j.apnu.2007.08.007 18809122

[76] Peckham E Allgar V Crosland S Heron P Johnston G Newbronner E Health risk behaviours among people with severe mental ill health during the COVID-19 pandemic: analysis of linked cohort data. *PLoS One.* (2021) 16:e0258349. 10.1371/journal.pone.0258349 34648548 PMC8516268

[77] Coventry P Brown J Pervin J Brabyn S Pateman R Breedvelt J Nature-based outdoor activities for mental and physical health: systematic review and meta-analysis. *SSM Popul Health.* (2021) 16:100934. 10.1016/j.ssmph.2021.100934 34646931 PMC8498096

[78] Sarkar C Webster C Gallacher J. Residential greenness and prevalence of major depressive disorders: a cross-sectional, observational, associational study of 94 879 adult UK Biobank participants. *Lancet Planet Health.* (2018) 2:e162–73. 10.1016/S2542-5196(18)30051-2 29615217

